# Ubiquitin C‐terminal hydrolase L1 (UCHL1), a double‐edged sword in mammalian oocyte maturation and spermatogenesis

**DOI:** 10.1111/cpr.13347

**Published:** 2022-10-11

**Authors:** Donghui Yang, Qizhong Lu, Sha Peng, Jinlian Hua

**Affiliations:** ^1^ College of Veterinary Medicine, Shaanxi Centre of Stem Cells Engineering & Technology Northwest A&F University Yangling Shaanxi China; ^2^ State Key Laboratory of Biotherapy and Cancer Center, Research Unit of Gene and Immunotherapy, Collaborative Innovation Center of Biotherapy, West China Hospital Sichuan University Chengdu China

## Abstract

**Background:**

Recent studies have shown that ubiquitin‐mediated cell apoptosis can modulate protein interaction and involve in the progress of oocyte maturation and spermatogenesis. As one of the key regulators involved in ubiquitin signal, ubiquitin C‐terminal hydrolase L1 (UCHL1) is considered a molecular marker associated with spermatogonia stem cells. However, the function of UCHL1 was wildly reported to regulate various bioecological processes, such as Parkinson's disease, lung cancer, breast cancer and colon cancer, how UCHL1 affects the mammalian reproductive system remains an open question.

**Methods:**

We identified papers through electronic searches of PubMed database from inception to July 2022.

**Results:**

Here, we summarize the important function of UCHL1 in controlling mammalian oocyte development, regulating spermatogenesis and inhibiting polyspermy, and we posit the balance of UCHL1 was essential to maintaining reproductive cellular and tissue homeostasis.

**Conclusion:**

This study considers the ‘double‐edged sword’ role of UCHL1 during gametogenesis and presents new insights into UCHL1 in germ cells.

## Graphical abstract

UCHL1 and mammalian gametogenesis. UCHL1 affects apoptosis‐related factors in mammalian gametogenesis and controls mammalian oocyte development, regulates spermatogenesis and inhibits polyspermy. It may also have other biological functions in the testis or ovary, such as anti‐inflammatory or antiviral.

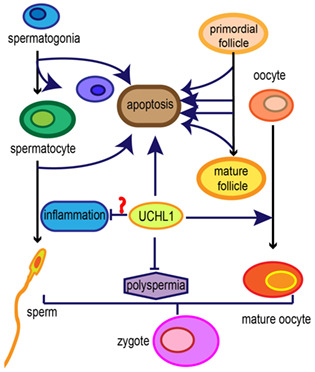

## 1 INTRODUCTION

Mammalian gametogenesis requires the precise expression of various enzymes and proteins, which is indispensable to the complex process to maintain its stability and balance. The functional integrity of proteins in the process of oocyte maturation and spermatogenesis is an important guarantee for sperm‐oocyte fusion. Amounts of studies have shown that ubiquitin proteasome system (UPS) can not only degrade misfolded or damaged proteins but also regulate protein expression levels to maintain a relatively stable state for mammalian gametogenesis.^1^, ^2^ Deubiquitination (DUB) is the reverse process of ubiquitination, which efficiently maintain the homeostasis of intracellular protein. UCHs family is one of the members of UPS, and it plays an important role in maintaining protein stability and balance. Although ubiquitin C‐terminal hydrolases L1 (UCHL1) has been extensively studied in many neurological diseases, such as Parkinson's disease^3^ and Alzheimer's disease,^4^ the mechanism in the reproductive process was not clear. In this review, we summarize the crucial roles of UCHL1 in mammalian oocyte maturation and spermatogenesis, to provide a reference in future studies about UCHL1 function in mammalian gametogenesis.

## 2 UBIQUITIN PROTEASOME SYSTEM

**FIGURE 1:**
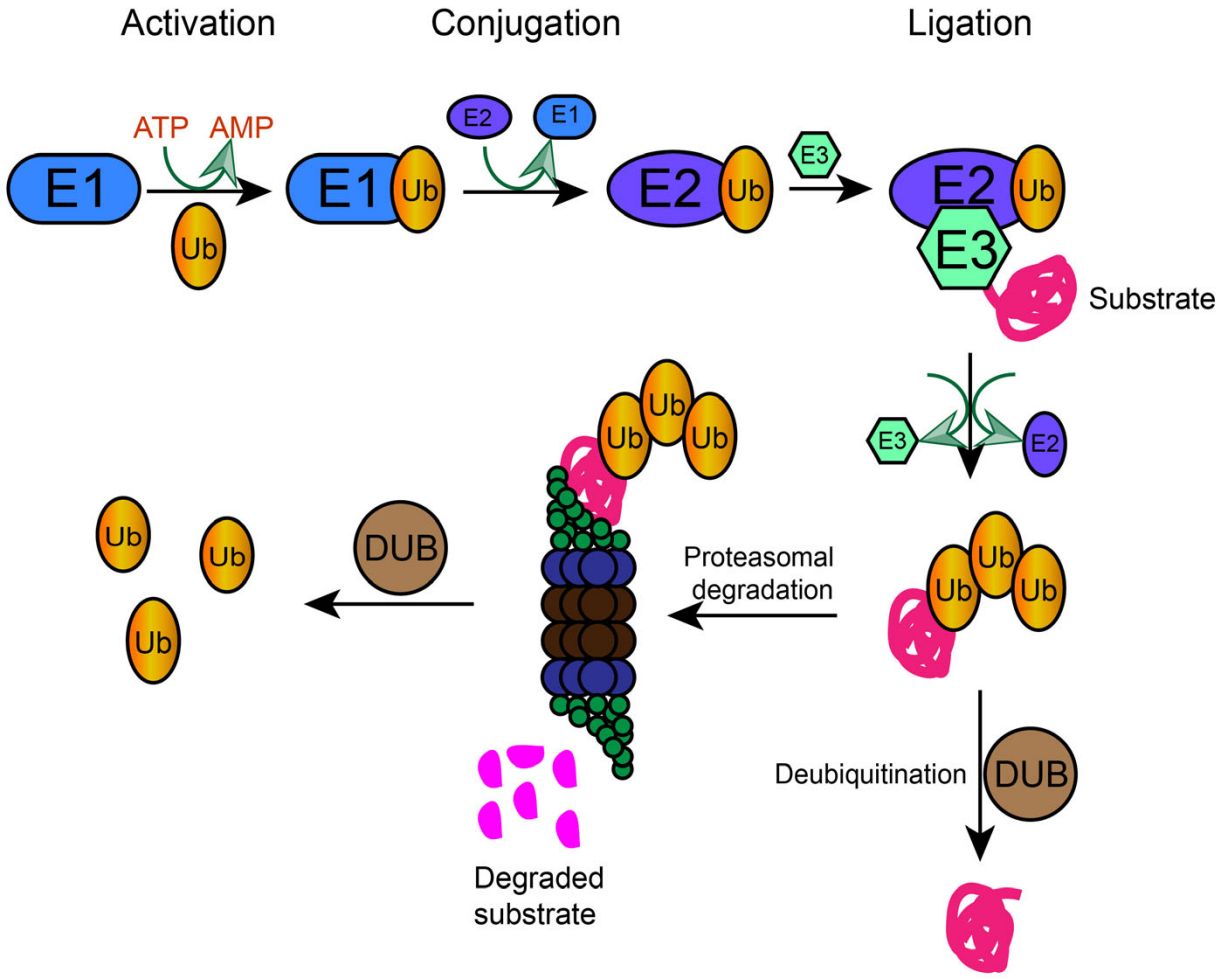
Ubiquitin proteasome system. It is the major pathway for intracellular protein degradation and is involved in more than 80% of intracellular protein degradation. The system includes Ub, ubiquitin‐activating enzyme E1, ubiquitin‐conjugating enzyme E2, ubiquitin ligase E3, 26S proteasome, and a series of DUBs. Proteins are first labelled by ubiquitin molecules and then recognized and degraded by the proteasomes. Cells degrade excess or incorrect proteins by consuming energy in a highly specific manner. DUBs, deubiquitinases; Ub, ubiquitin

The ubiquitin proteasome system (UPS) is primarily driven by ubiquitin (Ub) as a degradation tag, a process controlled by various reversible enzymatic reactions. It includes ubiquitin, ubiquitin‐activating enzyme E1, ubiquitin‐conjugating enzymes E2, ubiquitin‐ligase enzymes E3 and proteasome.^5^, ^6^ Ubiquitination of proteins is achieved by sequential activation of E1, E2 and E3 (Figure 1). E1 activates the ubiquitin molecule by forming a high‐energy thioester between its cysteine residue and the C‐terminal glycine residue of ubiquitin in a manner that consumes ATP.^7^, ^8^ Next, the activated ubiquitin molecule is transferred to E2, which interacts with E3, and then transfers the activated ubiquitin molecule to the ε‐amino group of the lysine residue of the target protein.^9^, ^10^, ^11^ Ubiquitin attaches to the substrate proteins by catalysing a series of covalent binding between the C‐terminus of ubiquitin and the internal lysine residues of the substrate.^10^ The proteasome is a multi‐subunit protease complex with a sedimentation coefficient of 26S, also known as the 26S proteasome. A typical 26S proteasome consists of a core hollow catalytic 20S subcomplex and a regulatory 19S subcomplex in a manner that consumes ATP. The 19S subcomplex recognizes, binds, and removes polyubiquitin chains attached to proteasomal degradation proteins.^12^ The polyubiquitin chains, consisting of ubiquitin and substrate, are recognized by the 26S proteasome, which breaks down the ubiquitinated proteins into short peptides, which are then released from the 20S core hollow, and finally, the polyubiquitin chains are decomposed into single molecules that re‐enter the ubiquitin cycle.^13^ UPS is involved in a variety of biological processes such as protein degradation, cell cycle regulation, stress response, DNA repair, immune response, transcriptional regulation, apoptosis, tissue remodelling, and development, etc.^14^, ^15^, ^16^, ^17^, ^18^ The dysfunction of UPS leads to an imbalance in intracellular protein homeostasis, which leads to the development and progression of various diseases.

Deubiquitination (DUB) is the reverse process of ubiquitination and is accomplished by a series of DUB enzymes. DUB enzymes hydrolyze ubiquitin molecules on target proteins and dissociate ubiquitin chains into individual ubiquitin molecules, playing a significant role in maintaining a dynamic balance between free ubiquitin molecules and those bound to target proteins (Figure 1). DUB enzymes not only deubiquitinate polyubiquitin proteins and recycle ubiquitin molecules removed by the 26S proteasome, but also remodel polyubiquitin chains.^19^, ^20^ The DUB process plays a role in repair and supervision in the UPS. DUB of proteins is involved in many biological processes, including cell growth and differentiation,^21^ transcriptional regulation,^22^, ^23^, ^24^ tumorigenesis and development.^23^, ^25^ Nearly 100 DUB enzymes have been identified in the human genome, which can be classified into seven types based on their catalytic subunits: ovarian tumour‐related proteases (OTUs), ubiquitin‐specific proteases (USPs), ubiquitin C‐terminal hydrolases (UCHs), Machado‐Joseph domain‐containing proteases (MJDs), motif‐interacting with ubiquitin containing proteases (MINDYs), JAMM/MPN domain‐associated Zn‐dependent metalloproteases (JAMMs) and zinc finger containing ubiquitin peptidase 1 (ZUP1).^26^, ^27^, ^28^, ^29^

Ubiquitin C‐terminal hydrolase (UCH) is an important member of the DUB enzyme family.^30^ It is a cysteine protease with a small molecular weight and a multifunctional protein. The main function of UCH is to catalyse the hydrolysis of peptide or isopeptide bonds, in detail, single ubiquitin or other proteins and ubiquitin groups in the polyubiquitin chains are conjugated with ubiquitin molecules to generate ubiquitin monomers. To date, the UCH family of DUBs consists of four main members: UCHL1, UCHL3, UCHL5/UCH37 and BRCA1‐associated Protein‐1 (BAP1).^31^ Currently, many studies are focusing on UCHL1 and UCHL3. UCHL1, also known as Protein Gene Product 9.5 (PGP9.5), is one of the most intensively studied UCH members.^32^ Previous studies have confirmed that UCHL1 plays an essential role not only in tumorigenesis, proliferation and metastasis but also in the nervous and reproductive system.^33^, ^34^ UCHL1 consists of approximately 220 amino acids and has a molecular mass of about 24.8 kDa. UCHL1 protein contains 223 amino acids in humans and mice,^35^ 220 amino acids in tilapia,^36^ and contains 218 amino acids in Zebrafish.^37^ Although the amino acid length of UCHL1 varies among species, its basic structure is highly conserved during evolution, and its conserved domain consists of 200 amino acids. UCHL1 is a multifunctional protein that has the activities of DUB, multiple ligases, and hydrolases, and participates in the ubiquitin‐mediated protein degradation pathway. Ligases mainly participate in the conjugation of the protein ubiquitin to a target protein, thereby, labelling that protein for recognition and destruction by the proteasome, and then dissociated. The main function of hydrolase is to hydrolyze short peptides or ester bonds attached to the carboxyl terminus of ubiquitin. UCHL1 is expressed in various tissues and organs, mainly expressed in the nervous system and testicular tissues,^38^, ^39^ with particularly high expression in the nervous system. UCHL1 maintains the activity of brain neurons and the memory function of the brain by removing abnormal proteins through the UPS and autophagy.^40^ Previous studies have focused on the role of UCHL1 in neurodegenerative diseases, particularly Parkinson's disease (PD) and Alzheimer's disease (AD).^3^, ^41^ However, as studies progressed, UCHL1 was found to be highly expressed in various cancers originating from different tissues, including the brain,^3^ lung,^42^ breast,^43^, ^44^ kidney,^45^ colon and pancreas,^46^ and prostate.^47^ In recent studies, UCHL1 has also been associated with severe acute respiratory syndrome coronavirus 2 (SARS‐CoV‐2), which has a potential role in mediating neurological complications after SARS‐CoV‐2 infection.^48^, ^49^, ^50^ In addition, UCHL1 is involved in spermatogenesis,^51^, ^52^ oocyte maturation,^53^, ^54^ and polyspermy^55^ during gonadal development and possibly in the fertilization process, however the exact mechanism remains unclear. The mechanism of UCHL1 in the mammalian reproductive system, especially during gametogenesis remains to be explored.

## 3 MAMMALIAN GAMETOGENESIS

**FIGURE 2:**
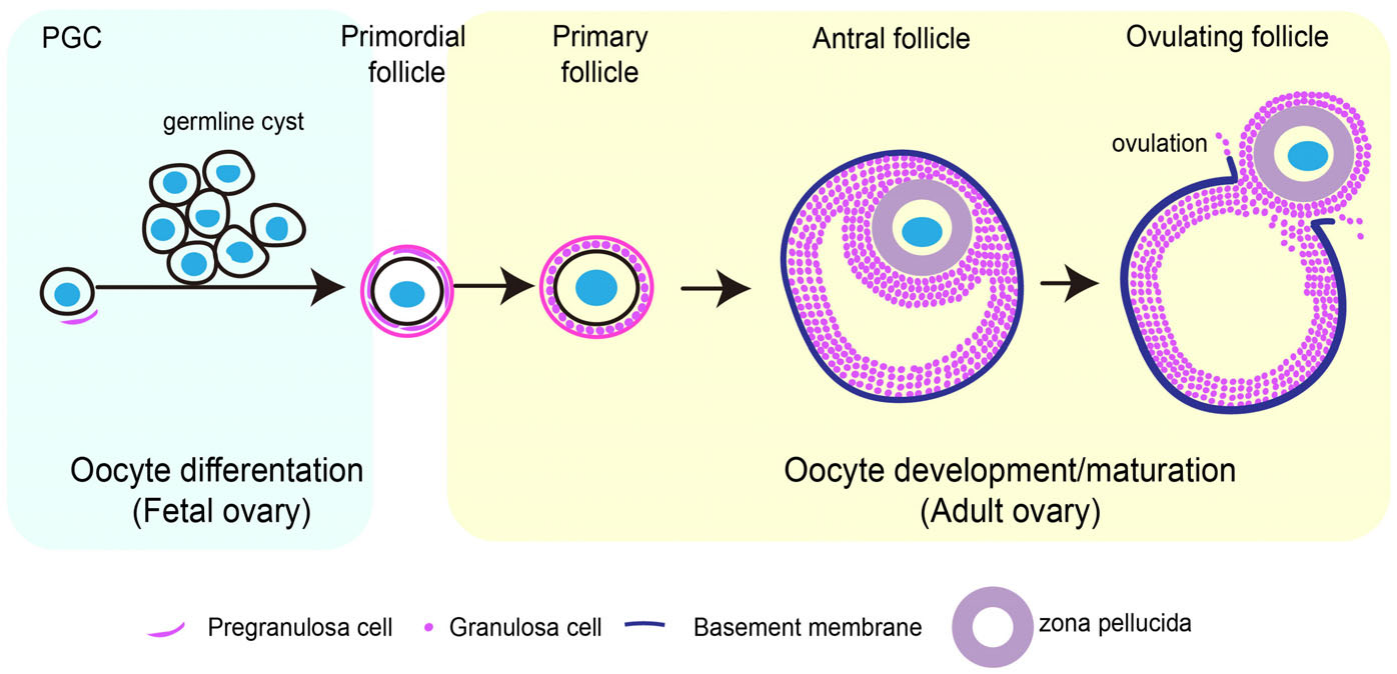
Mammalian oocyte development progress. Mammalian oocytes are derived from primitive germ cells formed in the foetal period. The entire process can be divided into two stages: oocyte differentiation and oocyte development. PGC migrates to the reproductive crest, proliferates, and differentiates into primordial oocytes by mitosis, which is encapsulated by a single layer of pregranulosa cells to form primordial follicles. During oocyte development, the primordium follicles develop into mature follicles through primary and secondary follicular stages, which eventually give rise to mature fertilized oocytes. PGC, primordial germ cell

Mammalian gametogenesis includes the development of sperm and oocyte, which are derived from primordial germ cells (PGCs). The formation of sperm and oocyte is a prerequisite for the formation of individual mammals.^56^ In male and female animals, PGCs differentiate into spermatogonia and oogenesis, and then, which enter the process of spermatogenesis and oogenesis, respectively, to form mature sperm and ovum. In female mammals, primordial follicles formed during the foetal stage are the only source of ovum production after the adult stage. Oogenesis can be divided into two stages: oocyte differentiation and oocyte development. During oocyte differentiation, PGCs differentiate into primordial oocytes, which are further encapsulated by a monolayer of pre‐granulosa cells to form primordial follicles. Primordial follicles eventually develop into mature follicles through primary and secondary follicles stages during oocyte development and eventually give rise to mature fertilized oocytes (Figure 2). The oocyte provides half of the nuclear genetic material for embryo formation and is endowed with almost all the cell membrane and cytoplasmic determinants required for embryo development.^57^ In addition to protein synthesis and modification by phosphorylation and dephosphorylation, selective degradation of certain specific proteins plays a key role in oocyte maturation. Moreover, the metabolic balance between oocytes and somatic cells ensures the availability of metabolites during oocyte maturation.^58^ The degradation of protein is essential for oocyte maturation.

**FIGURE 3:**
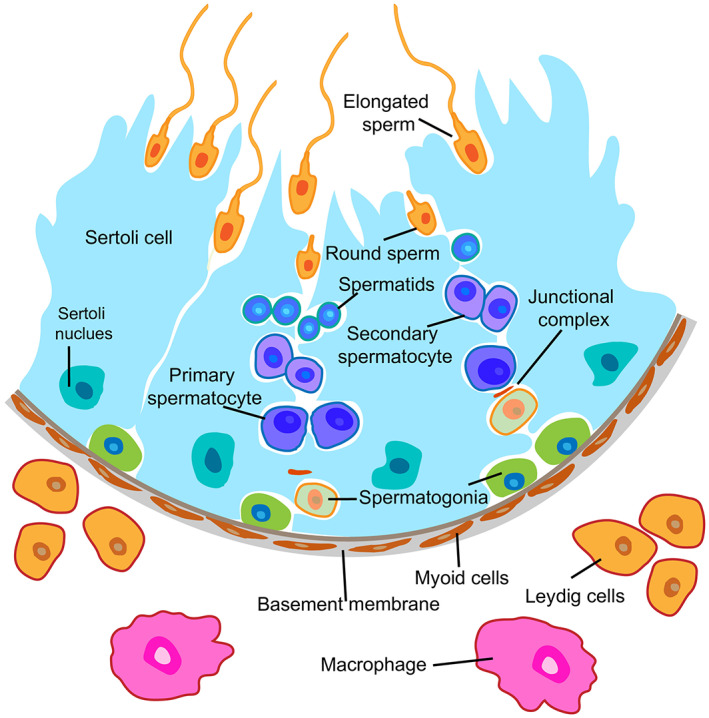
Mammalian spermatogenesis process. Spermatogenesis includes three stages, SSCs proliferation and differentiation, spermatocyte meiosis, and sperm deformation, respectively. Spermatogenesis involves not only germ cells but also other somatic cells. Such as Sertoli cells, Leydig cells, peritubular myoid cells, macrophages and other immune cells. SSCs and their surrounding environment are collectively referred to as stem cell niches. SSCs, spermatogonia stem cells

Spermatogenesis has a crucial role in controlling male fertility, and with the current advancements in various high‐throughput sequencing technologies, the integrity of the spermatogenesis process in different species has been further explained.^59^, ^60^, ^61^, ^62^, ^63^, ^64^ Spermatogenesis originates from SSCs localized to the basal membrane of seminiferous tubules, spermatogonia proliferate to form clusters of spermatogonia when they differentiate, which are connected by cytoplasmic bridges. Spermatogenesis is a highly organized, efficient, and coordinated process in which SSCs continuously proliferate, differentiate, and eventually produce maturation spermatozoa, which plays a key role in the continuity of the male reproductive system. The process of spermatogenesis in mammals can be divided into three sequential stages based on their functions: SSCs proliferation, spermatocyte meiosis, and spermatogenesis^65^ (Figure 3). Besides SSCs and spermatocytes, many surrounding somatic cells are also involved in spermatogenesis, such as Sertoli cells, Leydig cells, peritubular myoid cells, vascular cells, macrophages, and other immune cells.^66^, ^67^, ^68^, ^69^ In addition, spermatogenesis requires the participation of various hormones, paracrine factors, genes, and epigenetic regulators in the testis.^70^, ^71^ There are various post‐translational modifications involved throughout the development process from spermatogonia to spermatozoa. One of them is protein ubiquitination, which plays a role in the degradation of target proteins. Deubiquitination is the reverse process of ubiquitination and normal spermatogenesis must keep them in dynamic equilibrium.

Mammalian gametogenesis is regulated not only by gene transcription and translation but also affected by the degradation of various target proteins.^72^, ^73^ Indeed, UPS plays a regulatory and balancing role in mammalian gametogenesis. Additionally, ubiquitin proteins play a supervisory role in mammalian gametogenesis, in detail, they are responsible for the degradation of misfolded or damaged cellular proteins, and are also essential for controlling protein expression levels. The functional integrity of various proteins during gametogenesis is an important guarantee for the formation of zygotes. Several findings have demonstrated that UCHL1 is expressed in oocytes of humans and other primates,^74^, ^75^ as well as in some fish and amphibians such as toads.^36^, ^76^ However, UCHL1 is specifically expressed in spermatogonia of different animal testes, except in mice, where it is expressed in both spermatogonia stem cells (SSCs) and Sertoli cells.^51^, ^77^, ^78^, ^79^ At the same time, UCHL1 has been proved as a surface marker of SSCs in humans, monkeys, mice, cats, goats, pigs, cattle, and other mammals. Hence, it serves as one of the important molecular screening markers in vitro isolation and culture of SSCs from different species.^80^, ^81^, ^82^, ^83^ Taken together, UCHL1 is involved in gametogenesis or oocyte maturation and spermatogenesis in many animals.

### 3.1 UCHL1 controls oocyte development

Protein degradation is critical for oocyte maturation, and UCHL1 is one of the most abundant proteins in mammalian oocytes, and it is mainly expressed in the cytoplasm of primordial follicles and developing oocytes in human ovaries.^84^ Further research showed that high expression of UCHL1 was detected in monkey placental tissue, and the presence of UCHL1 in oocytes had the effect of inhibiting polyspermy, but the expression of UCHL1 was not detected in uterine tissue.^54^, ^74^ UCHL1 is mainly expressed in yolk in the ovaries of fish, and it is highly expressed during development and diapause, but hardly detected in the mature yolk, suggesting that UCHL1 is closely associated with the occurrence of yolk.^36^ tUCHL1 protein is highly homologous to mouse UCHL1 in oocytes of toads, and tUCHL1 may affect the resumption of meiosis in oocytes. These results indicate that tUCHL1 plays an important role in the maturation of amphibian oocytes.^76^ The development of porcine oocytes stagnated at the transition point of the middle and late periods of the first meiosis after inhibiting UCHL1. Meanwhile, UCHL1 is associated with upregulation of the activity of the histone H1 kinase relevant CDK1‐cyclin B complex and decreased ubiquitin levels,^85^ suggesting that UCHL1 is essential for the development of porcine oocytes. However, UCHL1 regulates the exclusion of the first polar body of oocytes and the localization of cortical particles thus affecting the maturation of oocytes in mice and rhesus monkeys.^58^ Numerous studies have shown that prostate tumour overexpressed‐1 (PTOV1) protein expression level increases gradually during ovarian maturation in mice, and specifically binds to UCHL1, they are involved in oocyte maturation and development under the action of oestrogen. Therefore, it plays an important role in cell cycle regulation.^86^ Additionally, UCHL1 is also associated with the apoptosis of oocytes. Several studies indicated that UCHL1 is expressed at higher levels in abnormal oocytes than in normal oocytes and changes in Jun activation domain‐binding protein 1 (Jab1) and p27^Kipl^ during oocytes formation in mice, suggesting that UCHL1 functions together with Jab1 and p27^Kipl^, and is synergistically involved in the selective elimination of mouse oocytes before sexual maturity.^87^ Recent studies have shown that UCHL1 expression is upregulated during the early proliferation and differentiation of mouse uterine decidua, creating a unique intracellular distribution pattern. Therefore, it was hypothesized that UCHL1 may respond to oestrogen regulation while participating in decidualization.^75^ Simultaneously, studies have shown that LDN‐57444, an inhibitor of UCHL1, could regulate oxidative stress, and mitochondrial function and reduce ERK1/2 expression, thereby inhibiting mouse oocytes' maturation.^53^ A recent study found that the loss of UCHL1 leads to decreased ovarian fertility and hormone responsiveness, severely affecting ovarian development and functional maintenance in mice.^88^

Oocyte maturation is considered as a physiological inflammatory response in a certain sense, accompanied by the participation of a variety of inflammatory factors. Studies have shown that supplementation with interleukin‐7 (IL‐7) enhances the meiotic maturation of porcine oocytes by improving both nuclear maturation and cytoplasmic maturation.^89^ Interleukin‐18 (IL‐18) induces cytokines interleukin‐1β (IL‐1β) and tumour necrosis factor‐α (TNF‐α) that are associated with follicle growth and oocyte maturation.^90^ It has also been found that deficiency of interleukin‐1 is associated with ovarian lifespan in mice.^91^ However, abnormal expression of inflammatory factors can trigger a pathological inflammatory response, which leads to the occurrence of ovarian diseases, such as polycystic ovary syndrome^92^ and endometriosis.^93^ Cancer usually accompanies the development of inflammation, and studies have shown that UCHL1 is associated with ovarian cancer.^94^ In conclusion, UCHL1 plays a significant role in oocyte development, and the changes in its expression during oocyte development seem to have different roles. However, the definite mechanism of UCHL1 remains to be explored, and whether it is associated with the DUB of UCHL1 is unclear.

### 3.2 UCHL1 regulates spermatogenesis

UCHL1, as a member of the DUB enzyme family in the UPS, is involved in the whole process of spermatogenesis. Therefore, disruption of UCHL1 homeostasis will lead to impaired spermatogenesis. For instance, the deletion of UCHL1 in mice shows a series of hindrances to spermatogenesis, such as abnormal morphology of seminiferous tubules, proliferation reduction of SSCs and spermatogenic dysfunction,^78^ suggesting that UCHL1 is indispensable in mammalian spermatogenesis. UCHL1 is expressed only in the testes in the gonads of many male mammals. Partial distribution of UCHL1 has also been detected in the epididymis of rats,^95^ humans^96^ and mice.^78^ The content in cauda epididymidis is higher than that in caput, the possible reason is that UCHL1 secreted from caput was absorbed by cauda. Studies have confirmed that the expression of UCHL1 in mouse testis has a time‐dependent effect. In detail, UCHL1 is only expressed in SSCs at 8–16 days after postnatal, and detected in both Sertoli cells and SSCs at 30 days after postnatal and adult mice.^51^ Recently, some researchers have used UCHL1 as a major molecular marker for human SSCs transplantation^97^ and porcine SSCs line establishment.^98^ However, UCHL1 is like a double‐edged sword, if its expression level is unbalanced, it will affect the normal physiological and biological processes. Studies have shown that transgenic mice are sterile if overexpressing UCHL1 because the meiosis process of primary spermatocytes was destroyed and led to spermatocyte apoptosis although the morphological and functional of Sertoli cells and spermatogonia are normal. At the same time, the UCHL1 staining of primary spermatocytes is weaker than spermatogonia and Sertoli cells, and the results of proliferating cell nuclear antigen (PCNA) detection indicated that the spermatogonia mitosis was blocked.^99^ These results suggest that overexpression of UCHL1 can seriously affect the formation of spermatocytes, and thus affect spermatogenesis. UCHL1 not only participates in germ cell apoptosis but also controls the factors that prevent apoptosis during spermatogenesis. Other studies have shown that the apoptosis levels of germ cells are positive‐related to increasing UCHL1 expression, which is possibly due to the DUB of p53 and the activation of mitochondrial apoptosis pathways.^100^ UCHL1 is involved in protein degradation during spermatogenesis, but protein ubiquitination can reduce cellular DNA damage during heat stress in SSCs. Additionally, germ cells are resistant to heat stress‐induced apoptosis in mice lacking UCHL1, whereas overexpression of UCHL1 will lead to spermatogenesis arrest due to apoptosis. UCHL1 is essential for normal spermatogenesis, and its level is closely related to the process of spermatogenesis, which may be due to the disturbed balance between ubiquitination and DUB leading to abnormal spermatogenesis.^100^, ^101^ It is worth exploring how the body controls the balance between ubiquitination and deubiquitination levels in testis, which will provide a theoretical basis for the final production of high‐quality sperm.

As research progresses further, UCHL1 plays other roles in the testis in addition to controlling apoptosis. Such as resisting viral infection and maintaining cell homeostasis. Gao et al. and other scientists showed that giant salamander iridovirus (CGSIV) infection can be reduced by UCHL1 in giant salamander testis, but its detailed mechanism remains to be further studied.^102^ Recent studies have confirmed that the loss of UCHL1 will affect the maintenance of SSCs homeostasis and metabolism in mouse testis, as well as its differentiation ability. It is speculated that the metabolic disturbance of SSCs caused by UCHL1 deletion affects the process of spermatogenesis, resulting in decreased fertility in male animals.^103^ While the relationship between UCHL1 and inflammatory responses has been well studied in other diseases, for example, researchers have found that IκB‐α stabilization and NF‐κB inactivation are closely associated with the DUB of UCHL1, demonstrating that UCHL1 may be a negative regulator of inflammatory response.^104^, ^105^, ^106^ However, whether it has an anti‐inflammatory function in the mammalian reproductive system still lacks reliable experimental evidence, especially how it plays role in male reproductive system infection needs to be further studied, this will also be a major challenge for future research on germ cell homeostasis.

### 3.3 UCHL1 inhibits polyspermy

Fertilization is an orderly and continuous process in which a fully mature oocyte is penetrated by viable sperm to ensure the rapid co‐development of male and female prokaryotes. Monosperm fertilization is mainly mediated by zona pellucida proteins consisting of ZP1, ZP2, and ZP3 and is critical for the initiation of growth and development.^107^ Polyspermy is the penetration of two or more spermatozoa into the oocyte, which eventually fails to develop into a zygote,^55^ and the cortical granules of the oocyte prevent polyspermy by modifying the extracellular zona pellucida proteins ZP1, ZP2 and ZP3 after fertilization.^108^ Previous studies have shown that the addition of two UCHL1 inhibitors to bovine oocyte cultures significantly increased the rate of polyspermy, suggesting that UCHL1 controls polyspermy during bovine sperm‐oocyte fusion,^55^ and that UCHL1 activity is involved in sperm acrosome function and anti‐polyspermy defence during pig fertilization.^109^ The knockout mice with UCHL1 showed a significantly higher rate of multiple spermatozoa entry and a significantly lower number of fetal births compared with normal mice, although the zona pellucida response process was normal.^54^ Therefore, the mechanism of function of UCHL1 in inhibiting multiple spermatozoa entry in mice remains unclear. It has also been shown that UCHL1 expression was reduced within 6 h of fertilization under heat stress conditions, had higher levels of oxidative stress than control, and showed multiple spermatozoa entry under high‐temperature conditions, but there was no difference in sperm penetration.^110^ Therefore, the multiple sperm entry into oocyte associated with high temperature during fertilization may be the result of reduced UCHL1 levels.

In mammals, the efficiency of in vitro fertilization (IVF) is relatively low compared to in vivo fertilization. In recent studies, it has been found that multiple sperm entry into eggs is one of the main causes of abnormal in vitro fertilization, and therefore low pregnancy rates, high abortion rates, high rates of malformed foetuses, or genetic disorders of the foetus can occur.^111^ Therefore, it has become particularly valuable to investigate the process of polyspermy and its molecular mechanism.

## 4 SUMMARY AND PROSPECTS

The ubiquitin‐proteasome is a large family and the balance between ubiquitination and DUBs precisely controls the dynamic levels of various proteins in spermatogenesis and oocyte development. The UCH family, as one of the key members of the UPS, plays an essential role in mammalian gametogenesis and fertilization. UCHL1 is closely associated with oocyte maturation and spermatogenesis, sperm‐oocyte union, and other processes. The roles of UCHL1 in oocyte maturation and spermatogenesis are distinct, and the expression levels of UCHL1 are positively and negatively determined by cell status and development, and we have supplemented the functions of UCHL1 in oocyte maturation and spermatogenesis (Table 1). Additionally, UCHL1 controls apoptosis‐related factors in mammalian gametogenesis and may have other biological functions, such as anti‐inflammatory or antiviral. However, the mechanism by which the UCH family regulates germ cell origin, fertilization, and early embryonic development remains unclear. It is instructive to resolve the role of UCHL1 in mammalian reproductive development for further research on protein homeostasis and the reproductive development capacity of germ cells. This will help us to better explore the protein turnover of the ubiquitin‐proteasome pathway during the dynamic transformation of germ cells and its ability to control the proliferation and differentiation of these cells, which is of great significance for further understanding of germ cell genesis and early embryonic development. Meanwhile, it can provide a basis for gamete formation and embryonic development in mammals, especially in economic animals, and lay a platform for the treatment of infertility in endangered animals.

**TABLE 1:** Functions of UCHL1 in oocyte maturation and spermatogenesis

Process	Function
1 Oocyte development	Affect the first meiosis Regulate the exclusion of the first polar body and the localization of cortical particles Regulate cell cycle Oocytes apoptosis Lead to decreased ovarian fertility and hormone responsiveness
2 Spermatogenesis	Germ cell apoptosis Control the factors that prevent apoptosis during spermatogenesis Involved in protein degradation during spermatogenesis Resist viral infection and maintain cell homeostasis Affect the maintenance of SSCs homeostasis and metabolism
3 Polyspermy	Inhibit polyspermy

## AUTHOR CONTRIBUTIONS

Donghui Yang and Jinlian Hua designed the study and wrote the manuscript. Qizhong Lu and Sha Peng made instructions and corrections to the language and content of the article.

## CONFLICT OF INTEREST

The authors declare that they have no competing interests.

## Data Availability

Data sharing is not applicable to this article as no new data were created or analyzed in this study.
